# Acid Ionic Liquids as a New Hardener in Urea-Glyoxal Adhesive Resins

**DOI:** 10.3390/polym8030057

**Published:** 2016-02-24

**Authors:** Hamed Younesi-Kordkheili, Antonio Pizzi

**Affiliations:** 1 Department of Wood and Paper Sciences and Technology, Faculty of Natural Resources, Semnan University, Semnan 35131-19111, Iran; 2 LERMAB-ENSTIB, University of Lorraine, Epinal 88000, France; antonio.pizzi@univ-lorraine.fr

**Keywords:** acidic ionic liquid, urea-glyoxal resin, catalyst, MALDI TOF, ^13^C NMR, DSC

## Abstract

The effect of acidic ionic liquid (IL) as a new catalyst on the properties of wood-based panels bonded with urea-glyoxal (UG) resins was investigated. Different levels of *N*-methyl-2-pyrrolidone hydrogen sulfate ([HNMP] HSO_4_ (0, 1, 2, 3 wt %)) were added to prepared UG resin. The resin was then used for preparing laboratory particleboard panels. Then, the properties of the prepared panels were evaluated. The structure of the prepared UG resin was studied by ^13^C NMR, and thermal curing behavior of the resin before and after the addition of IL was measured by DSC. Additionally, the main oligomers formed in the UG reaction were identified by matrix-assisted laser desorption/ionization time-of-flight (MALDI TOF) mass spectroscopy. The results indicated that IL can be used as an efficient catalyst for UG resin. The physicochemical tests indicated that the addition of [HNMP] HSO_4_ from 0 to 3 wt % decreased the pH value of the glue-mix, and the pH decreased on curing to the same level as urea-formaldehyde resins. The gel accelerated with increasing catalyst content and with the decreasing of the pH in the UG resin. The panels prepared with IL had higher mechanical strength and dimensional stability compared to those made from UG resins containing NH_4_Cl. Scanning electron microscope (SEM) micrographs showed that the panels prepared with ionic liquid presented low porous. DSC analysis showed that the addition of IL to the UG resin decrease the energy of activation of the curing reaction to render possible cross-linking. The MALDI TOF results indicated a preponderant linearity of the oligomers formed, implying a high energy of activation of curing for UG resins.

## 1. Introduction

With the introduction of a non-toxic aldehyde such as glyoxal as a substitute of formaldehyde, urea-glyoxal (UG) resins can be produced as green wood adhesives. Contrary to urea formaldehyde (UF) resins, UG resins are eco-friendly and present lower water absorption and thickness swelling [1,2]. Hence, UG as green adhesive seems to have good potential in wood-based panels manufacturing in the near future. 

UG resins as other thermoset aminoplastic resins need an acid medium to polymerize and cross-link [1]. It is for this reason that, in the previous studies, ammonium chloride (NH_4_Cl) was used as the usual acid catalyst in the preparation of panels bonded with UG resins [1,2]. So far, several research works were carried out on the importance of the use of catalysts in aminoplastic thermoset resins [3,4]. Among several types of catalysts, ammonium salts (such as NH_4_Cl and NH_3_SO_4_) are one of the most important and effective types of catalysts used for accelerating resin curing. Compared to other types of catalysts, NH_4_Cl is cheap, effective, free of annoying byproducts, and easy to use. The influence of NH_4_Cl on resin curing involves the release of hydrochloric acid (HCl), which brings the pH to very low values and speeds up the resin cure rate. In spite of the several advantages of NH_4_Cl as a thermoset resin catalyst, it also presents some disadvantages: (I) several bubbles form in the glue line due to the decomposition of NH_4_Cl in the hot press and consequent emission of ammonia gas, which reduces the mechanical strength of the resin and of the panels bonded with it; (II) furthermore, chlorine (Cl) is a toxic element and raises concerns of environmental pollution, especially in the case of green adhesives such as UG resins; (III) the residual acid left in the glue line as a consequence of the addition of an acid catalyst can increase the degradation of the cured resin in particleboards [3] as well as causing degradation by acid hydrolysis of the wood carbohydrates at the interface wood/adhesive. Thus, due to the defects of NH_4_Cl, and the green nature and low mechanical strength of the UG resins, it is imperative to find a nontoxic and eco-friendly high-quality catalyst. 

Conversely, ionic liquids (ILs), which have been widely promoted as “green solvents,” are attracting much attention for applications in many fields of chemistry and industry due to their chemical stability, thermal stability, low vapor pressure and high ionic conductivity properties [5]. Over the last few years, ILs have been popularly used as solvents for organic synthesis and also have been used as media for extraction processes [5]. Previous studies have indicated that ILs can also be used as an efficient catalyst in the synthesis of various materials. Among different types of ionic liquids used as catalysts, Brønsted acidic ionic liquids such as *N*-methyl-2-pyrrolidone hydrogen sulfate ([NMP]+HSO_4_^−^) has been reported as being most effective in terms of short reaction time and with excellent yields of the desired product [6]. Wang *et al.* (2011) indicated that ([NMP]+HSO_4_^−^) can be used as a substitute of various types of homogenous and heterogeneous catalysts due to its special properties such as being green, of low viscosity, non-volatile, recyclable and inducing no corrosion [7]. Additionally, Brønsted acidic ionic liquids are emerging as most promising alternatives for a wide variety of acid-catalyzed reactions, as they have useful characteristics of both solid acids and mineral liquid acids, and they can be designed to replace customary mineral liquid acids such as sulfuric acid and hydrochloric acid in chemical processes [6].

Regarding the type and the content of the catalyst directly affecting resin curing and the performances of final products [3], the aim of this study was the investigation of the effect of Brønsted acidic ionic liquids as a new resin catalyst on the physical and mechanical properties of the wood-based panels bonded with UG resins. It is important to note that the acid part of the IL obtained by its hydrolysis due to the increased temperature during hot pressing is the accelerator of resin hardening. The added advantage is that, as the hardened glue line cools, since none of the two parts of the IL are volatile, the almost neutral IL forms again, bringing the pH of the hardened adhesive to almost neutral again. The period of acid contact with the wood surface is very short, thus minimizing any hydrolysis damage to the wood surface carbohydrates [8]. The resultant strength of the panel is then unaffected. 

## 2. Materials and Methods

### 2.1. Preparation of UG Resin

The UG resin was prepared according to the method of Deng *et al.* (2014) [1]. Glyoxal (40%) was placed in the reactor, and the reaction pH was adjusted to 4–5 with a 30% NaOH solution. Subsequently, the first urea (U1) was added, and the mixture was then heated to 75 °C for 2 h. Then, the second urea (U2) was added, and the reaction mixture became a weak acid at pH 4–5 for 1 h at 75 °C. Lastly, the reaction mixture became a weak alkaline at pH 7.5 and cooled to room temperature ready for use. The U1/U2 weight ratio was 2:1. UG resins used for this study were prepared at G/U molar ratio of 1.4:1.

### 2.2. [HNMP][HSO_4_] Synthesis

**Figure 1:**
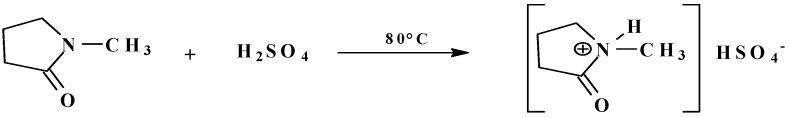
Synthesis of [HNMP] HSO_4_.

[HNMP] HSO_4_ was synthesized according to the method of Wang *et al.* 2011 [7]. 1.0 mol *N*-methyl pyrrolidone was weighed and placed into a 250 mL three-neck flask, and 1.0 mol sulfuric acid was added dropwise into the 250 mL three-neck flask with a dropping funnel under continuous mechanical stirring by magnetic stirrer at room temperature. A considerable amount of white smoke appeared during the dropwise addition of acid. The mixture became a sticky, light yellow transparent liquid after stirring at 80 °C for 4 h. The resultant liquid [HNMP] HSO_4_ was washed three times with ether and ethyl acetate, respectively, and was dried under vacuum to remove the ether and ethyl acetate. The schematic synthesis of [HNMP] HSO_4_ is shown in Figure 1.

### 2.3. Addition of Catalysts to UG Resin

Different proportions of [HNMP] HSO_4_ (0, 1, 2, 3 wt %) were added to prepare the UG resin at room temperature. To compare the performance of the acidic ionic liquid with NH_4_Cl, a UG resin containing 2 wt % NH_4_Cl (20% solution) was prepared as a control sample. These resins were then immediately used to prepare the wood panels. 

### 2.4. Measurements of the pH and Gel Time

The pH values of the resins at both ambient and elevated temperatures were measured with a Hanna pH meter (Limena, Italy). Before each measurement, the pH meter was calibrated with standardized buffer solutions at pHs of 4 and 7. After calibration, 200 g of a UG sample was pipetted into a 250-mL beaker, and the initial pH values of the resin and the solution, adjusted by the gradual addition of [HNMP] HSO_4_ from 0% to 3% (solids on solids), were recorded as ambient pH after 5 min of magnetic stirring at 20 °C. For measuring pH at hot condition, the temperature of UG resin increased to about 50 °C (before gelation state occurred), and ILs were then added to the resin. pH value was measured at this temperature as hot pH. In order to determine the gel time, five grams of the resin were introduced into a dry glass beaker. Then, aqueous acidic ionic liquid was added to the resin. The beaker was then immersed in boiling water, and the time until hardening was measured according to the Younesi-Kordkheili *et al.* (2015) method [9]. Two replicates for each sample were made.

### 2.5. DSC Analysis

The changes in enthalpy and curing temperature of the resins were determined using the thermal analyzer NETZSCH DSC 200 F3 model. The DSC scans were recorded at a heating rate of 10 °C/min under nitrogen atmosphere with a flow rate of 60 cm^3^/min. To determine the curing temperature of the resins, about 5 mg of freeze-dried sample was added to the aluminum pan. Additionally, 2% ILs was added to the prepared resins (based on dry weight) before measuring the curing temperature of resins. Then, the samples were heated from ambient temperature (25 °C) to 200 °C under nitrogen atmosphere.

### 2.6. CP-MAS ^13^C NMR Analysis

Solid-state cross-polarization/magic angle spinning ^13^C nuclear magnetic resonance (CP-MAS ^13^C NMR spectrum of the hardened resin after grinding to a very fine powder was recorded on a Brüker MSL 300 spectrometer at a frequency of 75.47 MHz. Chemical shifts were assigned relative to tetramethyl silane (TMS). The rotor was spun at 4 kHz on a double bearing 7-mm Brüker probe. The spectrum was acquired with 5 s recycle delay, a 90° pulse of 5 µs, and a contact time of 1 ms, and the number of transients was 3000.

### 2.7. MALDI TOF-MS Analysis

The samples were dissolved in acetone (5 mg/mL). 2,5-dihydroxy benzoic acid was used as the matrix. For the enhancement of ion formation, 0.1 M NaCl was added to the matrix. The solutions of the sample and matrix were mixed in equal amounts, and 1.5 μL of the resulting solution was placed on the MALDI target. After evaporation of the solvent, the MALDI target was introduced into the spectrometer. The spectra were recorded on a MALDI TOF instrument (AXIMA Performance, Shimadzu, Manchester, UK). The irradiation source was a pulsed nitrogen laser with a wavelength of 337 nm. The duration of a single laser pulse was 3 ns. The measurements were carried out using the following conditions: polarity-positive, flight path-linear, mass-high (20 kV acceleration voltage), and 100–150 pulses per spectrum. The delayed extraction technique was used by applying delay times of 200–800 ns.

### 2.8. Particleboard Manufacturing

The manufacturing of the particleboards was performed according to the method of Younesi-Kordkheili *et al.* (2015) [10]. A forming mold frame (35 cm × 35 cm × 1 cm) was placed on a stainless steel caul plate to control thickness. The surfaces of each plate were sprayed with a mineral oil releasing agent to ease demolding of the panel after hot pressing. Dried particles were then blended with the prepared resins in a rotating drum-type mixer fitted with a pneumatic spray gun. Then, compounded material was poured into the frame and spread to fill the frame evenly. When forming was complete, the top caul plate was placed on the top of the mat, and the entire assembly was placed into an oil-heated press that was used for compression molding. The temperature of the press plates was maintained at 180 °C at a maximum pressure of 25 bar pressure. The pressing of the compound material was carried out respectively in two stages by hot and cold presses for 5 min. A 5 cm wide edge of each board was trimmed to remove the low density and poor bonding areas of the boards [11]. To ensure reproducibility, three panels were manufactured for each formulation. The nominal thickness and density of manufactured panels was 16 mm and 0.8 g/cm^3^, respectively. Preparation of the samples for mechanical tests and water absorption measurements was started 24 h after pressing.

### 2.9. Panel Testing

All physical and mechanical tests of the particleboards prepared were carried out to the appropriate standard methods. The tests performed on the specimens were internal bond strength (IB-tensile strength perpendicular to the panel plane) [12], static bending [bending strength and modulus of elasticity (MOE)] [13] and water absorption and thickness swelling [14]. The samples were conditioned at a temperature of 23 ± 2 °C and a relative humidity of 60% ± 5% for two weeks. Five specimens were tested for each panel.

### 2.10. Scanning Electron Microscopy (SEM)

The morphology of the particleboards was examined using a scanning electron microscope (XL30) supplied by Semtech Limited (Neuchatel, Switzerland). The fracture surfaces of the specimens after the bending test were sputter-coated with gold before analysis. All images were taken at an accelerating voltage of 17 kV.

## 3. Results and Discussion

### 3.1. Effect of the [HNMP] HSO_4_ Content on the pH and Gel Time of the UG Resin

**Figure 2:**
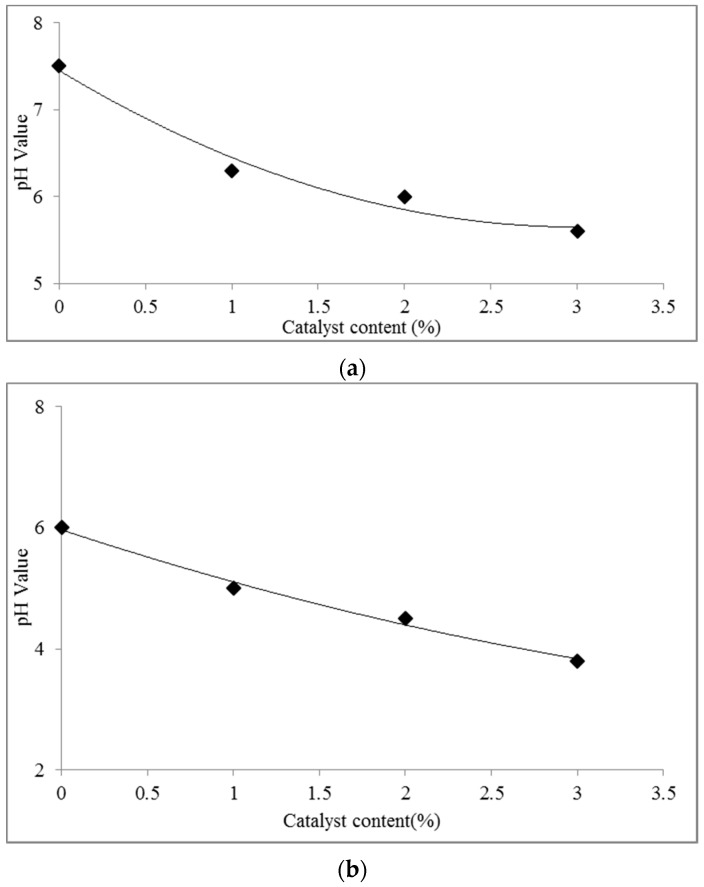
pH value of the UG resin *versus* the acidic ionic catalyst content at (**a**) ambient temperature (**b**) hot temperature.

The pH value of the UG resin decreased with increasing [HNMP] HSO_4_ content from 0% to 3%, as shown in Figure 2. Figure 2a showed that decrease in the pH value was initially very quick, and the slope of curve then became less sharp with increasing catalyst content. The effect of [HNMP] HSO_4_ on UG-resin curing is to release H^+^ and HSO_4_^2−^. With an increasing HSO_4_^2−^ concentration in the system, the rate of HSO_4_^2−^ release is retarded. Thus, the pH decreases very quickly in the beginning and then more slowly (Figure 2a).

According to Figure 2a, the curve of the UG resin containing 0%, 1%, 2% and 3% catalyst shows remarkable differences in pH values. This seems to contradict the previous findings about the influence of NH_4_Cl on UF resins. Previous studies have indicated that the pH value of UF resin decreases with catalyst addition very quickly at the beginning, but the changes in the pH value become smaller with increases in the catalyst content [4]. This finding indicates that, compared to NH_4_Cl, the use of acidic ionic liquid as a catalyst can decrease the pH value uniformly and continuously. Additionally, Figure 2b indicated that increasing ILs from 0% to 3% decreased the pH of resin at a hot temperature. This finding gives important information about the behavior of UG resin under a hot pressing condition for wood panel manufacturing. According to Figure 2b, the pH of the resin is still higher than 3.8, even with the addition of 3% catalyst. This is comparable to the pH obtained by catalyzed urea-formaldehyde resins, and it ensures that the pH reached on curing by the UG resin catalyzed with IL is not damaging to the lignocellulose of the wood substrate. 

**Figure 3:**
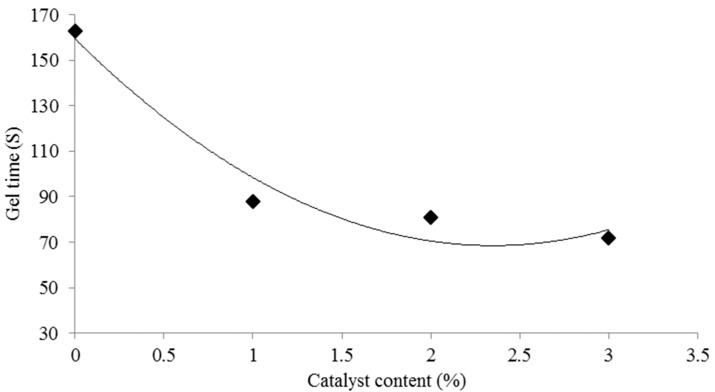
Effect of acidic ionic catalyst content on gel time of UG resin.

One of the most important resin parameters affecting the processing of wood-based panels is the resin gel time. The gel time is the time from when the material begins to soften to when gelation occurs, where gelation is the irreversible transformation from a viscous liquid to an elastic gel. The effect of the acidic ionic liquid content on gel time is shown in Figure 3. It can be seen that the addition of ILs from 1% to 3% significantly reduces the gel time of UG resins. The faster gel time of catalyzed UG resins indicates that their cross-linking rate becomes faster by adding [HNMP] HSO_4_. The shortening of the resin gel time with the addition of the catalyst is surely related to the decreasing pH value. The advantages of shorter gel times of thermoset resins with the addition of catalysts have been reported by several researchers [15,16,17]. 

### 3.2. DSC Results

**Figure 4:**
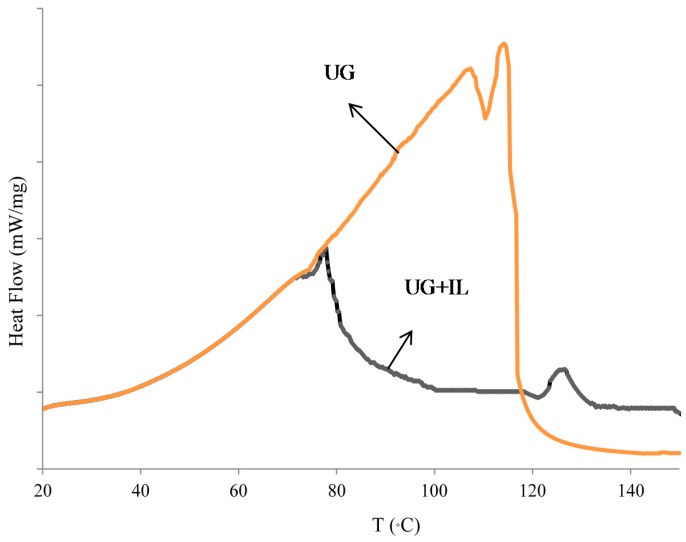
DSC curve of the UG resin and UG resin + 2% ILs.

The influence of ILs on thermal curing behavior of UG resins was investigated by DSC. The DSC curves of the UG resin without catalyst and UG resin with 2% ILs are shown in Figure 4. The curing of the UG resin is an exothermic reaction similar to other formaldehyde-based thermoset resins. The exothermic peak of the UG resin is affected by the presence of the ionic liquid. Adding the IL caused the peak of temperature (*T*_peak_) to be reached much earlier and at a lower peak temperature. For the pure UG resin, the average *T*_peak_ was about 103 °C. Adding the ILs did change the peak temperature to 77 °C. The decrease in peak temperature of the UG resin containing the IL catalyst indicated that ILs accelerates the hardening of a UG resin. The DSC analysis also showed that *T*_onset_ increased from 35 to 45 °C with the addition of 2% ionic liquid. The clear indications of all these are the addition of IL to the UG resin decreasing the energy of activation of the curing reaction, rendering possible cross-linking. This was confirmed by the DSC results showing that the enthalpy of the cure reaction (Δ*H*) (the area under the DSC exotherm curves) of the UG resin containing ILs was lower than that of pure UG, indicating also that low heat was generated by this mix.

### 3.3. ^13^C NMR of UG Resin

**Figure 5:**
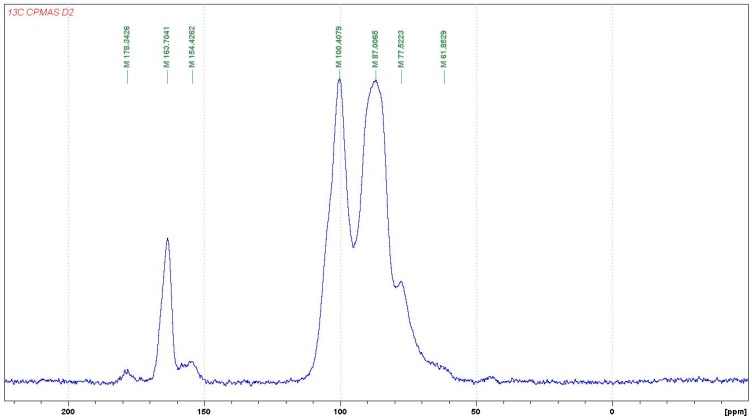
^13^C NMR spectrum of UG resin.

Previous studies have reported that the structure and property of thermoset resins vary as the synthesis conditions change [2]. Nuclear magnetic resonance spectroscopy (NMR) is one of the most effective methods of studying resin structure. So far, ^13^C NMR has been used to characterize the structures of various resins, such as UF, PF and PUF resins. The solid phase CP MAS ^13^CNMR spectra of a hardened UG resin is shown in Figure 5. The NMR of the hardened UG resin shows some features of interest. The shift at 163 ppm is indicative of the C=O of urea disubstituted for reaction with the glyoxal, and the shift at 154 ppm indicates a urea more multisubstituted, indicating tridimensional cross-linking of the hardened resin. The shifts at 87, 77 and 61 ppm are indicative of the carbon shifts of the following repeating unit,

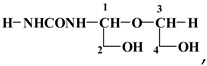

with the 87 ppm shift belonging to the C1 carbon directly linked to the –NH– group of urea, the 77 ppm shifts to the C3 not linked to the urea, and the wide but small 61–62 ppm peak belonging to the C2 and C4 of the repeating unit above.

The peak at 100 ppm belongs to the C linked to two urea molecules, as in the structure:

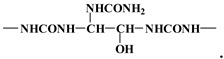


### 3.4. MALDI TOF Results

**Figure 6:**
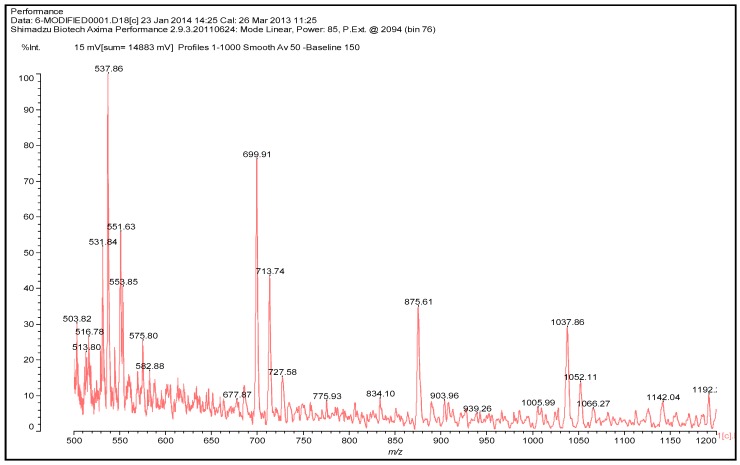
MALDI TOF spectrum of the UG resin.

The MALDI TOF spectrum in Figure 6 shows a number of peaks. There are clearly two repeating motives in the spectrum, one of 175 + 1 Da and a second one of 161 + 1 Da.

These are representative of the following two repeating units, namely for the 174–176 Da:

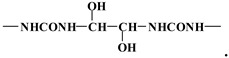


The second repeating mass interval at 162 Da is indicative of the following repeating unit:

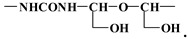


The two units are forced to alternate to form a repeating unit that is:

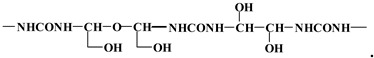


This alternance give rise to the series of peaks at 699–728, 875, 1037 and 1192 Da.

The following one in the series is:

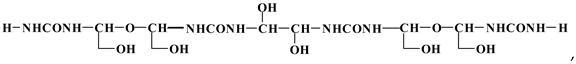

and so on. The peak at 538 Da is derived from the above oligomer species by the loss of a –CH_2_OH group and added to the Na+ used as a matrix enhancer. Thus, for example, the peak at 728 Da is the following molecule deprotonated and added to 23 Da of the Na^+^used as enhancer of the matrix

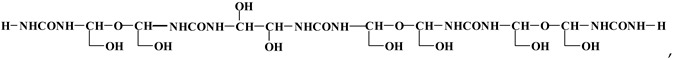

728 Da, deprotonated +Na**^+^**.

The peaks at respectively 699 and 713 Da are obtained from the above molecule by the loss of an –CH_2_OH and –OH group, respectively. The peak at 875 Da is derived from the addition of a 176 motive to the 713 peak to form an oligomer of the type

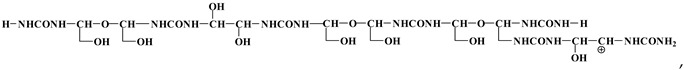

which, added to the Na^+^ used as a matrix enhancer, gives 875 Da. The following peak at 1037–1038 Da is the oligomer derived by adding a further 162 Da fragment to the 875 Da oligomer, giving the following type of oligomer:

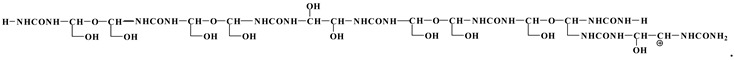


Three more findings result from the MALDI TOF analysis, the first of which being that the –CH–O–CH– ether bridges are formed by glyoxal resins just as easily as methylene ether bridges are formed in UF resins. These types of bridges, however, appear to be more stable than the methylene ether bridges of UF resins, where rearrangements with elimination of formaldehyde are rather common. However, rearrangements of such bridges with elimination of glyoxal cannot be excluded at this stage. These glyoxal ether bridges present in the 162-Da repeating unit alternate with definite –CH–CH– bridges characteristics of the 176-Da repeating unit. One last thing evident from the MALDI analysis is that the oligomers formed are mainly linear and appear to present little ramification, a fact that should definitely have a bearing on the gelling and curing time and behaviors of such adhesives. One of the bearings of these characteristics is most likely to be the higher energy activation for curing UG resins than that for UF resins. That is why ILs, and the lower pHs reached during hot-pressing, are necessary for proper curing.

As regards to the lower pH reached on curing, this has been shown to do little damage to the lignocellulosic substrate due to a very short period of time the acid is free and in contact with it, exclusively during the short hot pressing time, before being again neutralized on the cooling of the panel and the reforming of the IL in the cured resin [8]. Moreover, the pHs reached with the adhesive system UG+IL are not lower than what was achieved by a UF resin and a standard hardener.

### 3.5. Properties of Particleboards

**Figure 7:**
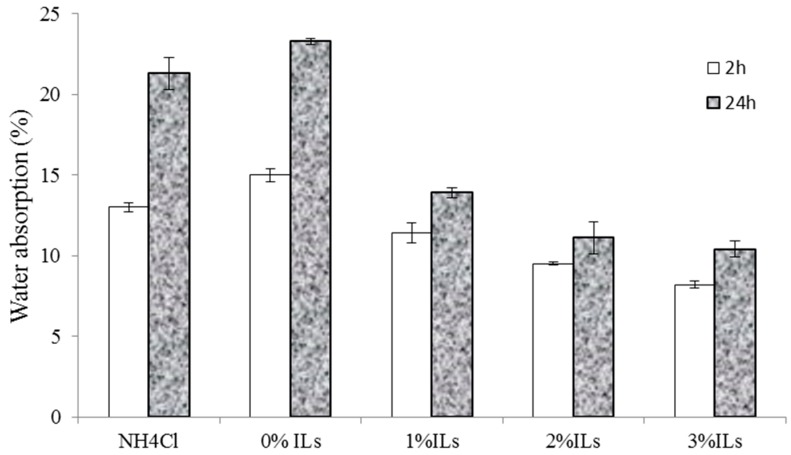
Water absorption of the manufactured panels made from synthesized resins.

**Figure 8:**
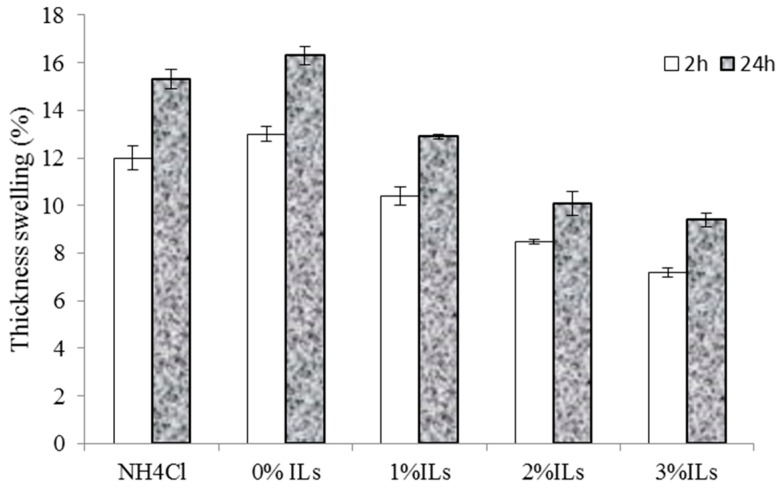
Thickness swelling of the manufactured panels made from synthesized resins.

Water absorption and thickness swelling of wood-based panels is one of the main factors that limit their outdoors application. As regards to this issue, several researchers have focused on finding new methods to reduce water absorption of wood-based panels [9,18]. Short-term water absorption and thickness swelling of the panels prepared are shown in Figure 7 and Figure 8, respectively. 

These (Figure 7 and Figure 8) show that water absorption and thickness swelling increase with increasing immersion time. The panels prepared without any catalysts exhibit the highest water absorption (23%) and thickness swelling (16%). Conversely, the panels containing acidic ionic liquid exhibit lower water absorption and thickness swelling compared to those catalyzed with NH_4_Cl. The results also show that the lower water absorption and thickness swelling content can be achieved by increasing the ionic liquid proportion from 0% to 3%, as shown by the panels containing 3% [HNMP] HSO_4_ exhibiting lower water absorption and thickness swelling. Higher water absorption and thickness swelling of the panels prepared with NH_4_Cl compared to those containing [HNMP] HSO_4_ can be explained by the SEM micrographs. SEM micrographs shown later indicate that, contrary to NH_4_Cl, the fracture surface of the panels prepared with the ionic liquid, produces no porosity and voids in glue line. These voids are the place where water molecules accumulate and increase the panel’s water absorption and thickness swelling. Conversely, it appears that the glue lines will be smoother and therefore less capable of absorbing water molecules in this region by increasing the acidic ionic liquid content. Kardos *et al.* (1986) also indicated that some microcracks and voids are created during the UF resins curing process [19].

**Figure 9:**
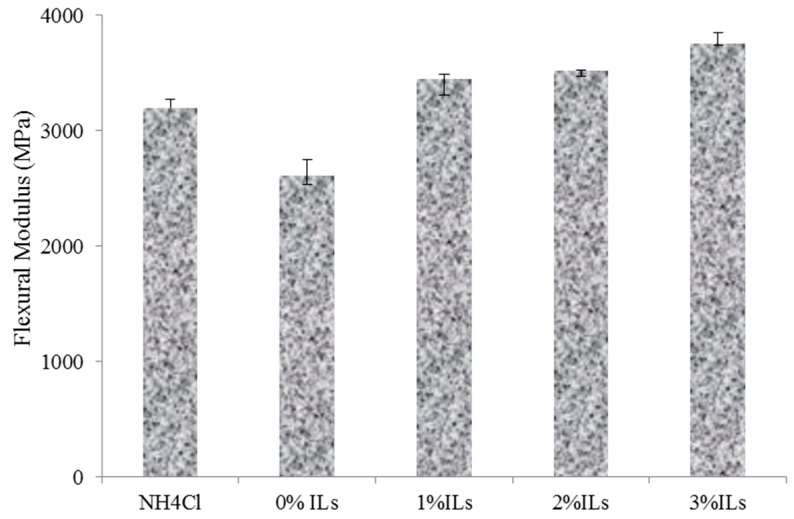
Flexural modulus of the manufactured panels made from synthesized resins.

**Figure 10:**
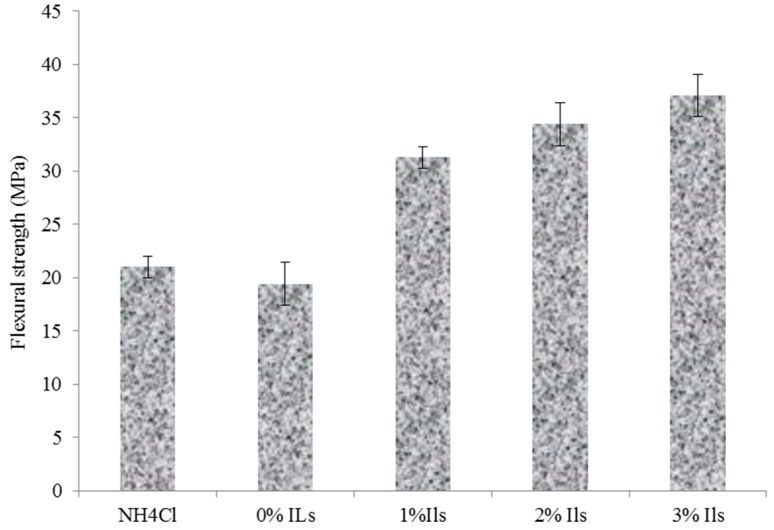
Flexural Strength of the manufactured panels made from synthesized resins.

The flexural modulus and strength of the panels containing 0%, 1%, 2% and 3% acidic ionic liquid compared to those had NH_4_Cl are illustrated in Figure 9 and Figure 10, respectively. It can be observed that panels with acidic ionic liquid exhibited higher flexural modulus and strength values than those that used ammonium chloride as a catalyst.

As shown in Figure 9 and Figure 10, increasing acidic ionic liquid content from 0% to 3% into UG resin improved flexural properties of the panels. The highest flexural modulus and strength values were related to UG resin contain 3% [HNMP] HSO_4_ as a catalyst, whereas the UG resin without catalysts exhibited the lowest values. In spite of the fact that glue line can act as a stress concentration point in bending strength, the higher MOE and MOR of the panels made from UG resin with acidic ionic liquid can be attributed to increasing mechanical properties of the resins with the addition of acidic ionic liquid. In addition, in hot press condition, the voids were produced after resin shrinkages or evaporation HCL gas in the UG resin structures. The voids in the glue line can reduce the mechanical properties of the resin as well as the panels. So far, several researchers have shown that a high amount of addition of NH_4_Cl decreases the mechanical strength of the resin and associated panels [20].

**Figure 11:**
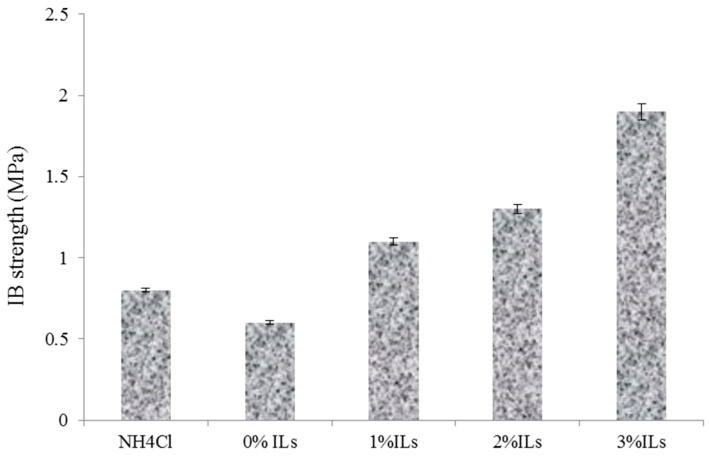
Internal bond strength (IB) of the manufactured particleboard made from synthesized resins.

Internal bond (IB) of the manufactured panels is shown in Figure 11. It can be seen that greater IB value can be achieved for the panels made from UG resin with the addition of catalysts. The panels made with 1%, 2% and 3% ionic liquid had 27%, 62% and 130% higher IB strength than the control samples (NH_4_Cl), respectively. The greater IB strength can be attributed to higher heat conductivity of the ionic liquid compared to wood particles and NH_4_Cl. This causes better polymerization of the resin in the board core layer. So far, several researchers have indicated that the use of materials with higher heat transfer properties can reduce the hot press time and increase IB strength of the panel [21]. Wang *et al.* (2014) investigated the influence of various types of catalysts on the synthesis of different materials [7]. They indicated that [NMP]+HSO_4_ exhibited the best catalyst results among various acidic ionic liquids because it has several advantages: (1) the IL [NMP]+HSO_4_ shows better catalytic activity and high yields; (2) the halogen free IL is more advantageous and the solvent free conditions for its use meet the greener aspects of catalysis; and (3) [NMP]+HSO_4_ can be easily recycled after separation.

### 3.6. Morphology Characteristics

**Figure 12:**
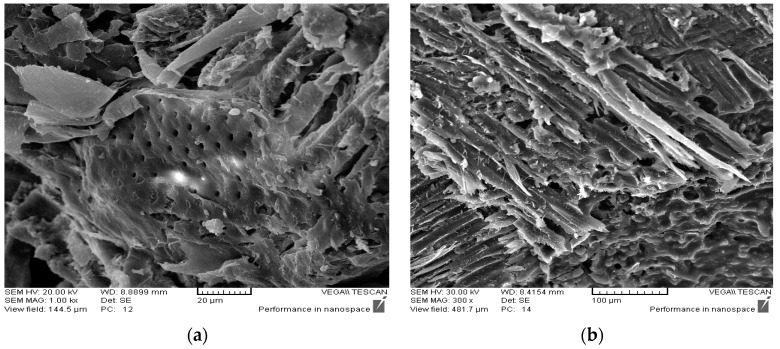
SEM micrograghs of the particleboards prepared with (**a**) UG resin+ NH_4_Cl; (**b**) UG resin+ ionic liquid.

Scanning electron microscopy is an effective media for the morphological investigations of wood-based panels. Figure 12a corresponds to the panels hardened with NH_4_Cl. As can be seen, there are bubbles in the fracture surface that cause weakness sites in the panels. Therefore, when stress is applied, it causes fractures to occur faster in this region. Contrary to the panels contain NH_4_Cl, there are no voids and bubbles in the particleboards with acidic ionic liquid hardeners (Figure 12b). Previous researchers have indicated that the existence of cavities in the wood-based panels helps the panels to absorb water and reduce mechanical properties. The panels, hardened with the green catalyst, present a smooth and monotonous matrix, and water penetration in the panels’ deeper holes and cavities is thus prevented. Previous studies have also shown that the high strength of the glue line directly influences the panels’ mechanical strength. 

## 4. Conclusions

In this work, the effect of an acidic ionic liquid ([HNMP] HSO_4_) as a new catalyst on physical and mechanical properties of wood-based panels bonded with urea-glyoxal (UG) resin was investigated. The following conclusions can be drawn from the results:

- Acidic ionic liquids, such as a halogen-free and ecofriendly ionic liquid, is an effective catalyst for urea-glyoxal resins.
- SEM micrographs showed that the wood-based panels containing an acidic ionic liquid had a smooth and monotonous matrix.
- The particleboard panels prepared with an ionic liquid hardener had higher mechanical strength and better dimensional stability compared to those made from UG resin containing NH_4_Cl.
- The gel time of the UG resin was accelerated with increasing [HNMP] HSO_4_ content.
- MALDI TOF mass spectroscopy indicated that UG resins need to overcome a high energy of activation on curing.
- DSC analysis showed that the addition of IL to the UG resin decrease the energy of activation of the curing reaction to render possible cross-linking.
